# Astaxanthin and thiamine dynamics in the copepod *Temora longicornis* in response to ultraviolet radiation exposure

**DOI:** 10.1371/journal.pone.0328379

**Published:** 2025-07-28

**Authors:** Samuel Hylander, Peter Sylvander, Rodrigo J. Gonçalves, Barbara Tartarotti, Thomas Roach, Emil Fridolfsson, Thomas Kiørboe, Pauline Snoeijs-Leijonmalm

**Affiliations:** 1 Centre for Ecology and Evolution in Microbial model Systems – EEMiS, Linnaeus University, Kalmar, Sweden; 2 Centre for Ocean Life, National Institute for Aquatic Resources, Technical University of Denmark, Charlottenlund, Denmark; 3 Department of Ecology, Environment and Plant Sciences, Stockholm University, Stockholm, Sweden; 4 Consejo Nacional de Investigaciones Científicas y Técnicas (CONICET) and Estación de Fotobiología Playa Unión (EFPU), Chubut, Argentina; 5 Department of Ecology, University of Innsbruck, Innsbruck, Austria; 6 Department of Botany, University of Innsbruck, Innsbruck, Austria; Central University of South Bihar, INDIA

## Abstract

Several aquatic top predators suffer from deficiency in vitamin B_1_ (thiamine), sometimes combined with low levels of carotenoid pigments, e.g., astaxanthin. The mechanisms leading to correlations between carotenoid pigmentation and thiamine status are not known. These substances and their precursors are produced by single-celled organisms and transferred to higher trophic levels via zooplankton. However, little is known about the factors regulating this transfer process and how it is affected by environmental stressors and zooplankton diet. We therefore exposed a common copepod, *Temora longicornis*, to ultraviolet radiation (UVR), which is an important environmental stressor, and to food items of different quality in terms of carotenoid profile. Astaxanthin was the most abundant carotenoid found in copepods. Its concentrations were negatively affected by UVR regardless of diet type, and the availability of an astaxanthin precursor (β-carotene) in the diet did not affect the response. Thiamine, on the other hand, showed a varying response, with elevated levels in copepods exposed to UVR at low β-carotene diet and lower levels in copepods exposed to UVR and high β-carotene diet. Altogether, this indicates that astaxanthin was consumed for photoprotection in the zooplankton and that thiamine dynamics might be modulated by UVR under certain dietary conditions. Hence, the concentrations of astaxanthin and thiamine in copepods are dynamic and to some extent regulated by exposure to UVR. Thus, the ability of zooplankton to transfer these substances to higher trophic levels depends, to some extent, on the exposure to environmental stressors.

## Introduction

All heterotrophic organisms need to ingest a number of essential compounds to survive and reproduce. Several important biomolecules such as fatty acids, vitamins, and antioxidants are produced mainly by single-celled organisms in aquatic systems. Animals are not capable of producing many of these essential molecules and are therefore dependent on dietary sources or provision of these compounds from their gut microbiota. Fatty acids have been relatively well studied but less is known about factors regulating the transfer and transformation of carotenoids and vitamins from phytoplankton via zooplankton to higher trophic levels [1–10].

The vitamin thiamine (vitamin B_1_) has recently gained interest in the scientific community [11]. The phosphorylated form of this water-soluble vitamin is an important co-enzyme in a number of central metabolic pathways [6]. Top predators including fish and birds in several ecosystems, e.g., in the Baltic Sea, the North American Great Lakes and the Pacific Ocean, have been reported to suffer from deficiency syndromes due to low levels of thiamine leading to a range of neurological symptoms and massive juvenile mortality [6,12–15]. These syndromes have in some cases also appeared in combination with low levels of carotenoids [16]. The reason for these correlations between carotenoids and thiamine are not understood, but could potentially develop if both substances are consumed due to oxidative stress. It has not been possible to relate thiamine deficiency and low carotenoid levels to contaminants or genetic factors, and a common hypothesis is therefore that the symptoms are caused by “large-scale environmental changes” that would affect the transfer of important biomolecules between trophic levels [6,10,13,17]. Several studies have addressed the production of thiamine and carotenoids in phytoplankton demonstrating that concentrations are affected by factors such as species composition and abiotic conditions [1–4,18–20]. Crustacean zooplankton (mainly copepods) are the main organisms in pelagic systems transferring and transforming thiamine and carotenoids to higher trophic levels such as fish [2–4,9,18,21]. However, little is known about which factors increase or reduce the efficiency of this transfer and to what extent zooplankton would transform these essential biomolecules [5,9,22].

Copepods (~ 1 mm in size) are some of the most abundant organisms in the mesozooplankton in both marine and freshwater habitats. They are able to selectively feed on phytoplankton with high nutritional value [23–25], which means that they are not passive conduits of what is available in the phytoplankton. Crustaceans, including copepods, are especially rich in the antioxidant carotenoid astaxanthin [9,26]. Only a few algal species produce astaxanthin directly; it is mainly synthesized by crustacean zooplankton from carotenoid precursors such as β-carotene and zeaxanthin [9,27]. Astaxanthin is a fat-soluble pigment with a chain of conjugated double-bonds and strong antioxidants, such as astaxanthin, are crucial in many organisms since they are involved in the reduction of reactive oxygen species (ROS) within cells. When ROS are produced in excess of the antioxidative capacity, they induce oxidative stress including degradation of macromolecules, proteins and membranes [9,28]. Astaxanthin is assumed to provide the copepods with an elevated capacity for a higher metabolism without increasing the oxidative stress [29,30]. External stress factors such as contaminants or exposure to ultraviolet radiation (UVR) can also increase the production of ROS and hence the oxidative stress [5,9,31]. UVR is an environmental stressor in all organisms and it has been shown to induce oxidative stress and DNA damage in zooplankton [32–34]. However, zooplankton employ a variety of defense mechanisms to reduce damage, e.g., by accumulating photoprotective compounds (such as astaxanthin) and inducing internal antioxidant defense systems [5,9,35]. Hence, astaxanthin has been suggested to be a multipurpose molecule in copepods with condition-dependent function, e.g., in photoprotection and as an antioxidant after UV-induced ROS formation [9].

The function and accumulation of thiamine in zooplankton is less studied [1–4,36] compared to astaxanthin dynamics [9]. This vitamin is an important substance in all organisms as it is cofactor of several central metabolic pathways and thiamine has, similarly to astaxanthin, antioxidant capacities [37,38]. Competition for thiamine has been suggested to be an important structuring force in aquatic systems at all trophic levels from bacteria to top consumers [6,10,39–41]. A series of recent laboratory and field experiments has shown that thiamine concentrations in zooplankton are species-specific but relatively stable over different seasons [1–4]. There is only a weak relationship between phytoplankton and zooplankton thiamine concentrations suggesting that zooplankton can maintain their thiamine pools during different conditions through selective feeding [1].

Hence, more information is needed on the transfer of astaxanthin and thiamine in the food web from lower to higher trophic levels. To disentangle factors regulating pigment and vitamin accumulation and transformation in zooplankton, we therefore conducted two mechanistic laboratory studies where we examined how changes in carotenoid profile of the diet affected the profiles of astaxanthin and thiamine in the zooplankton. We also exposed zooplankton to UVR stress to assess if oxidative stress changes the accumulation of pigments and vitamins. We used a copepod species from the Temoridae family, which is a functionally important and widely distributed family worldwide, with documented astaxanthin content [18,42,43]. We hypothesize that the accumulation of astaxanthin is related to UVR exposure and the availability of precursors in the food source, and that thiamine concentrations increase when astaxanthin is low as an alternative antioxidant when challenged by UVR.

## Materials and methods

### Experimental setup and culture conditions

**Fig 1:**
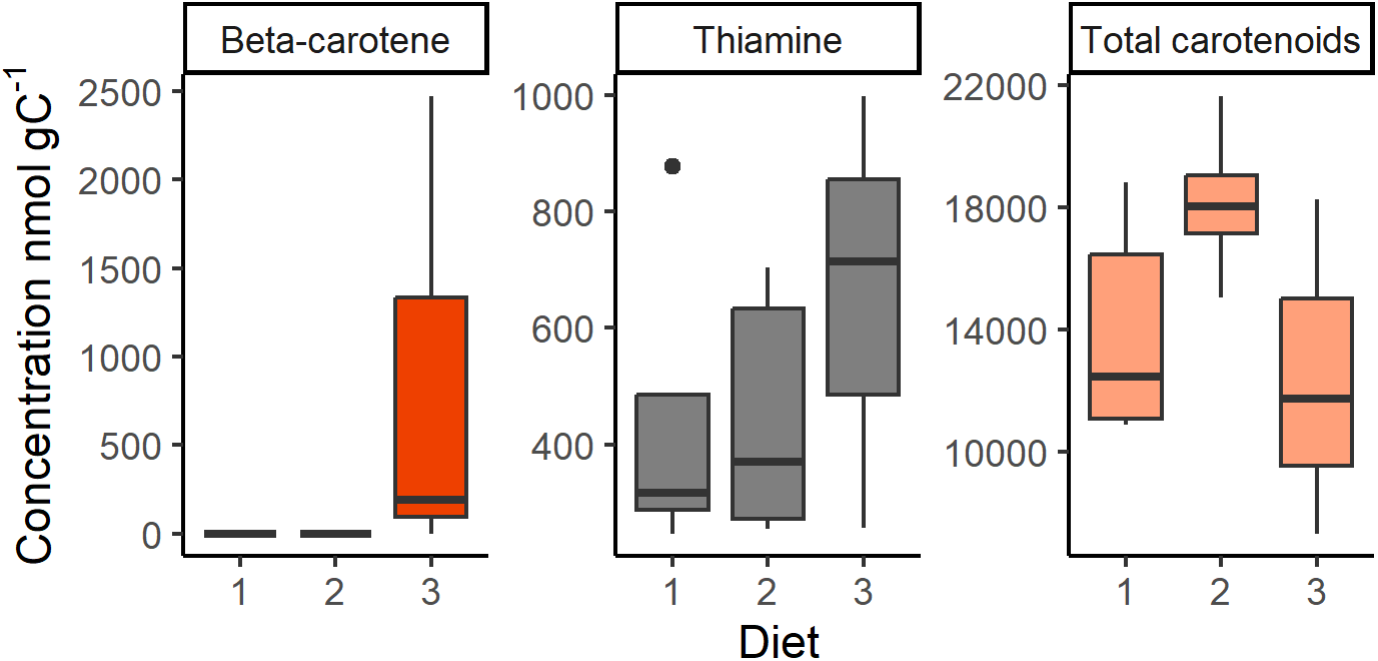
Diets. Concentrations of β-carotene (left), thiamine (middle) and total carotenoids (right) in Diets 1-3 used in the feeding experiments.

Temora longicornis, a common copepod species originating from the North Sea (Kattegat), were harvested from cultures kept continuously at 14 °C in dim light conditions and filtered sea water from the same area (0.2 µm, salinity 27) and fed an algal mixture (60% *Rhodomonas salina*, 10% *Heterocapsa triquetra*, 10% *Prorocentrum minimum* and 20% *Thallassiosira weissflogii*) three times a week ad libitum. The copepods for experiments were acclimatized seven days to a diet of *R. salina*, grown at 18 °C and approximately 80 µE m^-2^ s^-1^ in B1 medium prepared according to [44]. Two separate experiments were performed with three types of diets (see diets below; Fig 1). Experiment #1 (Exp 1) used Diet 1 and Diet 2, whereas Experiment #2 (Exp 2) used Diet 3. Both experiments were performed in plastic aquaria filled with 9–10 L pre-filtered (0.2 µm) seawater (same as above) with a salinity of 27 psu and a temperature of 20 °C. The copepods were concentrated with a 300 µm net to obtain a mixture of adults and copepodids (nauplii were not included) and we then added ca. 600 copepods per aquarium. Using indoor experiments, copepods were then either UV-exposed (UV) or not exposed (No UV) and provided with three different diets at saturating levels (Diet 1–2, Exp 1; Diet 3, Exp 2) with four (Exp 1) and five replicates (Exp 2) per treatment. Both experiments lasted for 11 days (Exp 1: May/June 2012 and Exp 2: May 2014).

### Diets with differential carotenoid and thiamine mixtures

To achieve different feeding conditions with respect to the carotenoid content, three different diets were used in the experiments (Fig 1). Diets 1 and 2 consisted of 100% *R. salina* grown under different light conditions (25 µE m^-2^ s^-1^ and 150 µE m^-2^ s^-1^, respectively; B1 media), whereas Diet 3 consisted of 50% *R. salina* and 50% *Dunaliella tertiolecta* (150 µE m^-2^ s^-1^; f2-media). The latter species is known to be a β-carotene producer, i.e., the main precursor used by crustaceans to produce astaxanthin [45–47]. β-carotene was absent in Diets 1 and 2 but present at median concentration of 299 nmol gC^-1^ in Diet 3 (Fig 1a). Zeaxanthin is also known to be a precursor for crustaceans to produce astaxanthin [9,46], but this carotenoid only occurred sporadically in concentrations below 0.6 nmol g C^-1^. Other carotenoids also occurred in the diets and their concentrations and relative proportions are available in supplementary material (Fig S1 in S1 File).

*Rhodomonas salina* does not produce thiamine [48], whereas *D. tetrilolecta* is considered to be a potential thiamine producer [48,49]. However, phytoplankton can also take up thiamine from the growth media regardless of potential thiamine auxotrophy [3,4]. The three diets contained thiamine concentrations ranging from 247−998 nmol gC^-1^. There were no significant differences in thiamine concentrations among diets (F_2,10_ = 0.47, p = 0.637; Fig 1b). The relative proportions of different thiamine vitamers in the diets is available in Fig S2 in S1 File.

Food levels were adjusted to 1000 µg C L^-1^ every second-third day after measurement of cell concentrations with a particle counter (Beckman Coulter Counter) or under a microscope (Carl Zeiss inverted light microscope, Primo Vert). The carbon content per cell was based on literature data [50].

### UVR treatment

Ultraviolet radiation is known to induce elevated oxidative stress in organisms [51,52]. To generate this oxidative stress, each aquarium was illuminated with two fluorescent lamps (36 W, UVA-340, Q-Panel) mounted 0.5 m above the aquaria with an 18:6 light:dark cycle, producing an intensity in the UVA spectrum of 523 ± 5.6 µW cm^-2^ (UVR sensors SUL 240, connected to a IL 1400A logger, International light, Newburyport, Massachusetts, USA). These fluorescent lamps are commonly used to simulate solar radiation in the UVR wavelength range [53,54]. For the complete spectrum of the lamps see Hansson et al. (2007) [53]. For differential UVR exposure, two different kinds of Plexiglas were used. In No UV treatments, the UVR radiation was blocked by using Plexiglas which cuts off radiation below 370 nm, i.e., the UVA and UVB range (Röhm GS 233®). In treatments exposed to UVR, radiation was transmitted by UVR-transparent Plexiglas (Röhm GS 2458®). There was no difference in transmittance of photosynthetically active radiation (PAR) between the two types [53]. To maintain good water quality, ~ 30% of the water was exchanged three times a week (clean, filtered water was added) and once a week the aquaria were rinsed.

### Sampling

Carotenoid, thiamine, and carbon concentrations in phytoplankton cultures (*R.* *salina* and *D. tetrilolecta*) used to feed zooplankton were sampled every second to fifth day. From each culture, 10–20 mL was collected on glass fiber filters (Whatman® GF/F; pre-combusted at 500 °C for 5 h for the carbon samples). All filters were stored at −80 °C and analyzed with High Performance Liquid Chromatography (HPLC) (see below). At the start of the experiments and after 11 days of exposure, 20–50 copepods were collected per aquarium for carotenoid and thiamine analyses and approximately 10 copepods per sample for carbon analysis. The animals were placed in filtered seawater (0.2 µm) for more than two hours prior to sampling to allow for gut evacuation and hence avoid interferences during the analysis from phytoplankton pigments originating from the digestive tract of copepods. There were not enough copepods for all analyses in some replicates. Hence, thiamine and astaxanthin estimates in copepods are missing for two replicates (UV1 Diet3 and UV3 Diet3, respectively). The copepods were then collected on glass fiber filter as above and stored at –80 °C until analysis.

### Pigment analysis

The carotenoid content of *R. salina* in Exp 1 was analyzed using HPLC according to Wright *et al.* (1997) [55] with a solvent gradient as described by Pinto *et al.* (2003) [56] . The filters were sonicated for 1 min in 100% HPLC grade methanol on ice (Vibra Cell™, amplitude 92, pulse 0.9 s), and centrifuged at 4 ºC for 4 min at 6000 × g. Unfortunately, material was lost during sonication of one replicate (No UV Diet1) not allowing for astaxanthin estimate in that replicate. The supernatant was filtered through a 0.45 μm pore size PTFE/PP filter (Titan™). HPLC was carried out using an Agilent™ 1100 System equipped with a scanning UV diode array detector and a SphereClone™ column (250 × 4.60 mm, 5 μm particle size, ODS2). The extracts were automatically diluted to 80% with 0.5 M ammonium acetate buffer immediately before 100 μL was injected into the system. Carotenoid extraction from *T. longicornis* in Exp 1 was conducted in the same manner as for *R. salina*. In this case, 75 μL of the extracts were injected into a Gemini C18 (Phenomenex™) column (30 × 3.00 mm, 3 μm particle size, 110Å) with a flow rate of 0.3 mL min^-1^ and a solvent gradient according to Snoeijs & Häubner (2013) [18]. For the analysis of pigments in Exp 2, freeze-dried copepods or algae were finely ground with the glass fiber filters using 2 x 5 mm quartz beads for 2 min at 30 Hz. For extraction, 100 and 500 µl of HPLC grade methanol were added to algae and copepods, respectively, and shaken for an additional two min, before centrifugation at 29,000 g and 4 °C for 40 minutes. For most samples, 50 µL of supernatant were injected into an Agilent 1100 HPLC system and pigments were separated using a LiChrospher 100, RP-18 endcapped LiChroCART 125−4 125 × 4.0 mm with 5 µm particle size column (Supelco Analytical, Supelcosil™), with a solvent gradient of acetonitrile:methanol (74:6) and methanol:hexane (5:1) at a flow rate of 1.0 mL min^-1^. Detection was via diode array detector at 440 nm and 650 nm for carotenoids and chlorophyll, respectively. Astaxanthin occurs in several forms, here categorized as mono-, di-esters or as E or Z isomers.

### Thiamine and elemental analysis

All thiamine extractions and analyses were conducted according to Pinto *et al.* (2002) [57]. Filters were sonicated for 1.5 min in 0.1 M HCl on ice (Vibra Cell™, amplitude 92, pulse 1 s), and centrifuged at 10 ºC for 15 min at 21000 × g. The supernatants were then mixed with 50 µL freshly made K_4_Fe(CN)_6_, 300 µL 1 M NaOH and 550 µL 100% HPLC grade methanol and vortexed and centrifuged once more at 10 ºC for 15 min at 21000 × g. The supernatant (600 μL) was then filtered through a 0.45 μm pore size PTFE/PP filter (Titan™) and analyzed by means of fluorescence in an Agilent™ 1100 system (Exp 1) equipped with a Reprosil-Pur™ NH2 column (5 μm particle size, 250 mm × 4.6 mm [I.D.]) from Coricon™ (Knivsta, Sweden) and a Hitachi Chromaster HPLC system (Exp 2) equipped with Purospher®Star NH2 LiChroCART ® column (5 μm particle size, 4.6 mm × 250 mm [I.D.]), protected by a Purospher ®Star NH2 LiChroCART ® guard column (5 μm particle size, 4 mm [I.D.] × 4 mm). An excitation wavelength of 375 nm and an emission wavelength of 450 nm were used. Injection volume was 100 μL and the flow rate was 1.0 mL min^-1^. Chromatograms were integrated using the software Chemstation (Agilent™) and three forms of thiamine were detected: free thiamine (TF), thiamine monophosphate (TMP) and thiamine diphosphate (TDP). Samples for elemental composition were dried at 60 °C for 24 h and then analyzed on a CHNS/O element analyzer (PerkinElmer® 2400 Series II).

### Statistical analyses

All statistical analysis and graphics were produced using RStudio 2021.09.0. Vitamin and pigment concentrations were analyzed with analysis of variance (ANOVA) with astaxanthin or thiamine concentration as y-variables and the factors radiation (No UV vs. UV), diet and their interactions as x-variables. Data were log-transformed to meet the assumptions of the tests. The relative amounts of different types of thiamine and carotenoids were analyzed with PCA (library factoextra) and using log-transformed data. Astaxanthin concentration in one replicate in the No UV Diet3 treatment was approximately 4.6 times higher than the highest value of all other replicates. Such a high concentration is very rare in marine copepods [9]. Given the large deviation from all other replicates it was regarded to be an outlier and was removed from all further analyses.

## Results

### Total astaxanthin in *Temora*

**Fig 2:**
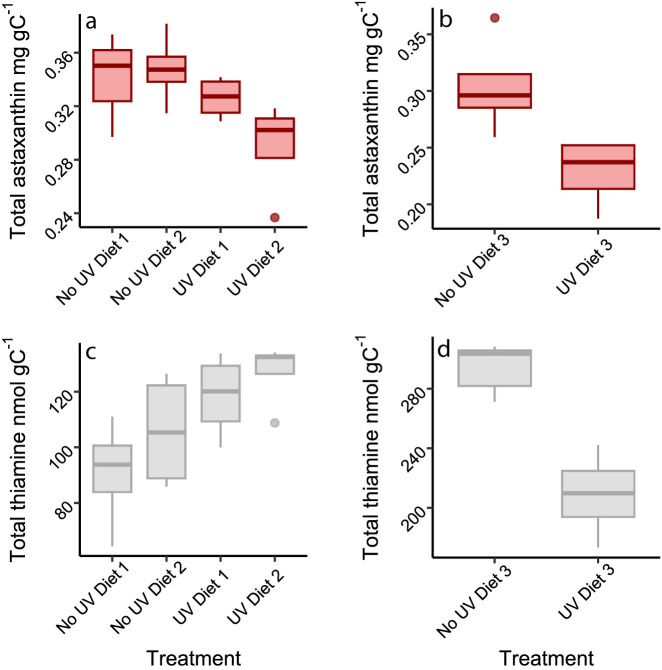
Astaxanthin and thiamine in copepods. The upper panels show the concentrations of astaxanthin with and without exposure to UVR in the copepods using Diets 1 and 2 (a) and Diet 3 (b), whereas the lower panels show thiamine concentrations with and without exposure to UVR in the copepods using Diets 1 and 2 (c) and Diet 3 (d). Panels a and c are from Exp 1 and panels b and d from Exp 2. Center line of the box denotes the median.

Copepods had 0.53 ± 0.11 and 0.46 ± 0.11 mg gC^-1^ (i.e., mg astaxanthin per g carbon weight) total astaxanthin at the start of Exp 1 (Diet 1–2) and Exp 2 (Diet 3), respectively (average ± SD, supporting information in S2 File). Copepods fed with Diets 1–2 had approximately 10% lower astaxanthin concentrations in UV compared to No UV treatments after incubation (Fig 2a; Table 1, F_1,11_ = 5.0, p = 0.047). However, there were no effects of diet or diet by radiation interactions on copepod astaxanthin (Table 1). When using Diet 3, astaxanthin concentrations were approximately 25% lower in the UV compared to No UV treatment (Table 1, t = 2.9, p = 0.029, df = 6). Hence, exposure to UVR consistently led to lowered levels of total astaxanthin in *Temora* regardless of diet used.

**Table 1:** Statistical results (ANOVA) on differences in concentrations of total astaxanthin and thiamine in copepods using Diets 1 and 2 (top) and Diet 3 (bottom). The term “Radiation” explains the UV versus No UV treatment effect, whereas the term “Diet” tests effects of Diets 1 and 2 in Exp 1.

Experiment #1 – Diets 1–2				
	Variable	df	F value	p-value
**Astaxanthin**	Radiation	1	5.0	**0.047**
	Diet	1	1.2	0.301
	Radiation:Diet	1	2.1	0.179
	Residuals	11		
**Thiamine**	Radiation	1	7.4	**0.018**
	Diet	1	1.7	0.212
	Radiation:Diet	1	0.2	0.631
	Residuals	12		
Experiment #2 – Diet 3				
	**Variable**	**df**	**t-value**	**p-value**
**Astaxanthin**	Radiation	6	2.9	**0.029**
**Thiamine**	Radiation	7	5.1	**0.001**

### Total thiamine in copepods

Copepods had 157 ± 37 and 386 ± 43 nmol gC^-1^ total thiamine at the start of Exp 1 and 2, respectively (average ± SD). Thiamine concentrations were approximately 25% higher in UV treatments compared to No UV treatments in Diets 1–2 after incubation (Fig 2c; Table 1, F_1,12_ = 7,4, p = 0.018). There was no effect of diet type or interaction between diet and radiation (Table 1). On the other hand, when using Diet 3 there was an opposite effect with 41% lower thiamine concentration in UV treatments compared to No UV treatments (Fig 2d; Table 1, t = 5.1, p = 0.001, df = 7). Hence, thiamine increased in response to UVR using Diets 1–2 but decreased when applying Diet 3.

### Relative proportion of carotenoids and thiamine vitamers in copepods

**Fig 3:**
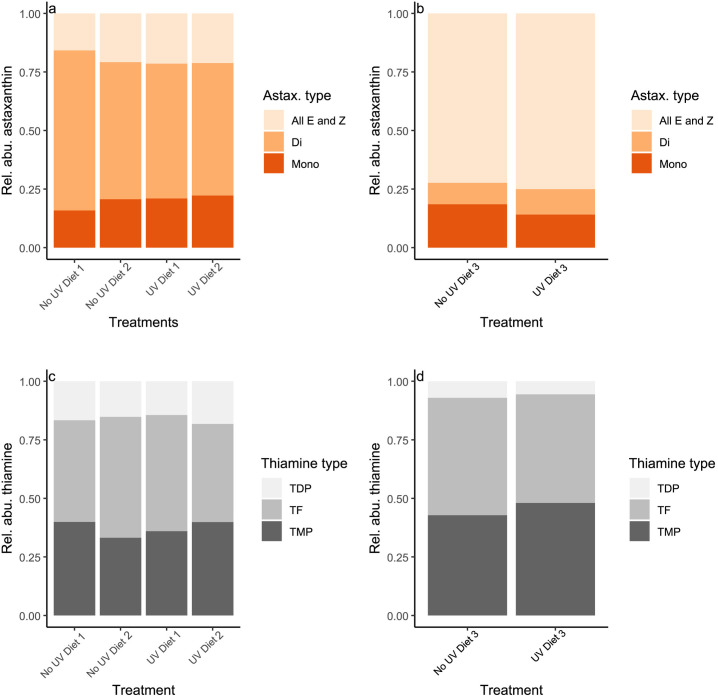
Relative proportions of astaxanthin and thiamine. The relative contribution of different astaxanthin and thiamine compounds in the copepods using Diets 1 and 2 (a, c) and Diet 3 (b,d). Astaxanthin occurs in several forms, here categorized as E or Z isomers, or as mono- or di-esters. Thiamine were detected in three forms including: free thiamine (TF), thiamine monophosphate (TMP) and thiamine diphosphate (TDP).

Most of the detected carotenoids in *T. longicornis* consisted of astaxanthin and there were only small amounts of chlorophyll and alloxanthin. Among the astaxanthin, di-esters and all E and Z isomers dominated the astaxanthin profile in copepods in Diets 1–2 and 3, respectively (Fig 3a). The mixture of the different astaxanthin types was relatively similar among treatments in both experiments, but No UV Diet 1 had a slightly different profile mainly driven by higher contribution of di-esters (Fig S3a in S1 File). Likewise, the mixture of different astaxanthin was similar between UV and No UV treatments using Diet 3 but with some differences, e.g., slightly lowered mono-esters, upon UV exposure (Fig S3c in S1 File).

The thiamine occurs in different forms (see Methods) and the profile in copepods was dominated by the vitamer TF followed by TMP and TDP (Fig 3c and 3d). The mixture of vitamers was not dramatically affected in Diets 1–2 but there was slightly less TMP in the No UV Diet 2 compared to UV Diet 2 treatment. In Diet 3 there were differences in mixture of vitamers with more TF and TDP and less TMP in the No UV compared to the UV treatment (Fig 3c and 3d, Fig S3d in S1 File).

## Discussion

The present results indicate that UVR exposure reduces and alters the relative contributions of astaxanthin and thiamine to the total pool of these compounds in zooplankton. Thus, the production and transfer of astaxanthin and thiamine in *Temora* is dynamic and their concentrations are regulated by environmental stressors. Here specifically, we have shown that exposure to UVR, which is an environmental stressor causing oxidative stress [9,32,34,51,52], reduces levels of the carotenoid astaxanthin in a pelagic copepod, suggesting that astaxanthin is consumed for photoprotection. Other studies have likewise observed lowered astaxanthin levels when copepods are subjected to UVR under starvation [58]. Our copepods were not starved but supplied with *R. salina* (Diets 1–2) or a mixture of *R. salina* and *D. tetrilolecta* (Diet 3). Hence, Diets 1–2 mainly contained the carotenoids alloxanthin and monodoxanthin. These carotenoids are generally not regarded as important astaxanthin precursors. Antajan and Gasparini (2004) [59] suggested that alloxanthin in copepod body tissues could be an intermediary pigment leading to further conversion to astaxanthin [59]. Alloxanthin is indeed a possible pathway to astaxanthin production in gold fish [60]. We detected some alloxanthin in copepod tissues (0.09 ± 0.07 mg gC^-1^, average ± SD in all samples), suggesting that this pathway may be present in the studied copepod species. Diet 3 contained β-carotene, which is a known precursor for astaxanthin conversion in crustaceans [9,27]. The concentration of β-carotene in Diet 3 was of the same order as found in natural phytoplankton communities [61]. However, this did not compensate for the loss of astaxanthin in the copepods and as in Diets 1–2, copepods had lower astaxanthin concentrations in UV compared to No UV treatments when fed the β-carotene-containing diet (Diet 3).

Interestingly, several studies in freshwater lakes have shown that copepods can increase their astaxanthin content when exposed to UVR, suggesting that stressed individuals accumulate/produce more pigments compared to non-stressed individuals [62–64]. Pigmentation (i.e., astaxanthin) increases the animals’ resistance to UVR since it is a strong antioxidant, alleviating damage from UV-induced ROS [65,66], but makes them more conspicuous to visual predators such as fish [64,67]. Copepods in clear lakes devoid of fish generally have high pigmentation and it has been suggested that this is an adaptation where astaxanthin acts as a neutralizer of harmful photoproduced radicals [9,68–70]. However, astaxanthin is also present in winter when the UV threat is low, indicating that the molecule is also important for other antioxidant protection purposes [30,71]) and may increase the overall metabolic capacity [9,29]. The copepods in the present study had astaxanthin concentrations within the normal range for marine copepods [9]. Hylander et al. (2014) [72] showed that marine copepods generally have lower astaxanthin concentrations than freshwater copepods, and this observation has recently been confirmed in a meta-analysis [9]. The mechanisms behind these differences are not known, but the present results indicate that astaxanthin dynamics in relation to UVR are different between marine and freshwater copepods.

The relative mixture of astaxanthin in the copepods was different in Diets 1–2 compared to Diet 3, but only slightly affected by treatment. The relative contribution of different astaxanthin esters is known to be related to ontogenetic stage and may also vary with zooplankton species composition [18,64,73]. There is also a seasonal component to the proportion of different esters [18]. The copepods in the present study were all adults or late copepodids and of the same species, demonstrating that diet and UV exposure, apart from ontogeny, season and species, can be factors that alter the relative contribution of different astaxanthin types in copepods.

Thiamine concentrations in copepods were either higher or lower in UV compared to No UV treatments, and all estimated concentrations fall within the range previously observed in marine copepods [1–4]. Likewise, thiamine concentrations in the diets were all within the range observed in natural communities and in laboratory studies [1–4,19]. It seems that the type of diet modulates the effect of UVR on the thiamine concentrations in copepods. Thiamine is an important cofactor in several metabolic processes, but can also act as an antioxidant [38], and one could hypothesize that zooplankton up-regulate other oxidative stress defenses (such as thiamine) when astaxanthin is low (as in Diets 1–2). It has previously been shown that zooplankton up-regulate their endogenous antioxidant systems, such as glutathione S-transferase (GST), when astaxanthin levels are lowered [70]. Several defense systems, including cell repair systems and internal antioxidant defenses, are employed when zooplankton are exposed to UVR [5,74], which could alter the metabolism of the animal and hence the requirements and concentrations of thiamine. The relative contribution of different types of thiamine to the total pool was mainly affected by Diet 3. The bioactive phosphorylated form (TDP) was lowered under Diet 3 and UV exposure, which may indicate lowered metabolism under UV exposure. Thiamine is crucial in metabolic processes in organisms [37], but also has antioxidant properties [38], and these different roles may explain the variable responses to UVR observed with different diets, but further studies are needed to pin down the mechanisms involved.

UVR defense mechanisms may also have been modulated by differences in food quality. The main difference between Diets 1 and 2 was that Diet 1 contained approximately 10% higher concentrations of alloxanthin and monodoxanthin. Diet 3 additionally contained the astaxanthin precursor β-carotene. Alloxanthin has been suggested to be accumulated at slow rates in the present copepod species [59], and we did detect some alloxanthin in the copepods. β-carotene, on the other hand, has been shown to be converted to astaxanthin in crustaceans [9,27]. Another potential difference in food quality between diets could be due to different light treatment of the phytoplankton (as in Diets 1–2), which may increase the accumulation of other antioxidants such as vitamin E [75] and affect the fatty acid composition of the phytoplankton [76]. Therefore, we cannot conclude if some other food quality component allowed the copepods to increase thiamine concentration in Diets 1–2, but could not maintain thiamine status in Diet 3.

In this mechanistic study, we conclude that UVR exposure, which is an important oxidative stressor, negatively affected astaxanthin concentrations and either positively or negatively affected thiamine concentrations. The relative contributions of different astaxanthin and thiamine compounds to the total pool of these substances were also slightly affected by the treatment. Overall, this suggests that astaxanthin can be lowered when used for UV protection, whereas thiamine can be mobilized or lowered during UV exposure. Thus, the production and transfer of astaxanthin and thiamine in copepods is dynamic and the concentrations of these substances are regulated to some extent by environmental stressors. Future studies should elucidate the molecular underpinnings of these responses to better understand how astaxanthin and thiamine dynamics are regulated at cellular and organismal levels. This includes the abiotic factors, such as light, that affect astaxanthin precursors in the diet, and the molecular mechanisms by which zooplankton accumulate or consume astaxanthin and thiamine. It would also be interesting to include other species adapted to a range of different UV exposures to be able to generalize the response and to use the results in aquaculture settings to promote more nutritious zooplankton. Finally, zooplankton are nodes in the food web as key prey items for fish and it is essential to understand how UVR exposure and interactions with climate change affect their status and nutritional profile.

## Supporting information

S1 File Contains supplementary figures: Fig S1. Relative proportions of carotenoids in Diet 1 (a) Diet 2 (b) and Diet 3 (c), and the total carotenoid concentration (d). Fig S2. Relative proportions of thiamine vitamers in Diet 1 (a), Diet 2 (b) and Diet 3 (c). Fig S3. Illustration of mixtures of astaxanthin in copepods using Diets 1–2 (subpanel a) and Diet 3 (subpanel c). (DOCX)

S2 File Raw data. All raw data in separate excel file.
(XLSX)
