# Supplementary material for: Astaxanthin and thiamine dynamics in the copepod Temora longicornis in response to ultraviolet radiation exposure
Source: PLoS One. 2025 Jul 28;20(7):e0328379. doi: 10.1371/journal.pone.0328379 (PMC12303292; doi:10.1371/journal.pone.0328379)
Supplement: S1 File — (DOCX) [file pone.0328379.s001.docx]

Supplementary material S1:

Astaxanthin and thiamine dynamics in the copepod *Temora longicornis* in response to ultraviolet radiation exposure

Samuel Hylander^1,2*^, Peter Sylvander^3^, Rodrigo J. Gonçalves^2,4^, Barbara Tartarotti^5^, Thomas Roach^6^, Emil Fridolfsson^1^, Thomas Kiørboe^2^, Pauline Snoeijs-Leijonmalm^3^

^1.^ Centre for Ecology and Evolution in Microbial model Systems – EEMiS, Linnaeus University, SE-39182 Kalmar, Sweden

^2.^ Centre for Ocean Life, National Institute for Aquatic Resources, Technical University of Denmark, DK-2920, Charlottenlund, Denmark.

^3.^ Department of Ecology, Environment and Plant Sciences, Stockholm University, SE-10691 Stockholm, Sweden.

^4.^ Estación de Fotobiología Playa Unión (EFPU) and Consejo Nacional de Investigaciones Científicas y Técnicas (CONICET), Argentina.

^5.^ University of Innsbruck, Department of Ecology, Technikerstraße 25, 6020 Innsbruck, Austria

^6.^ University of Innsbruck, Department of Botany, Sternwartestraße 15, 6020 Innsbruck, Austria

*Corresponding author: Samuel Hylander. [samuel.hylander@lnu.se](mailto:samuel.hylander@lnu.se); Centre for Ecology and Evolution in Microbial model Systems – EEMiS, Linnaeus University, SE-39182 Kalmar, Sweden


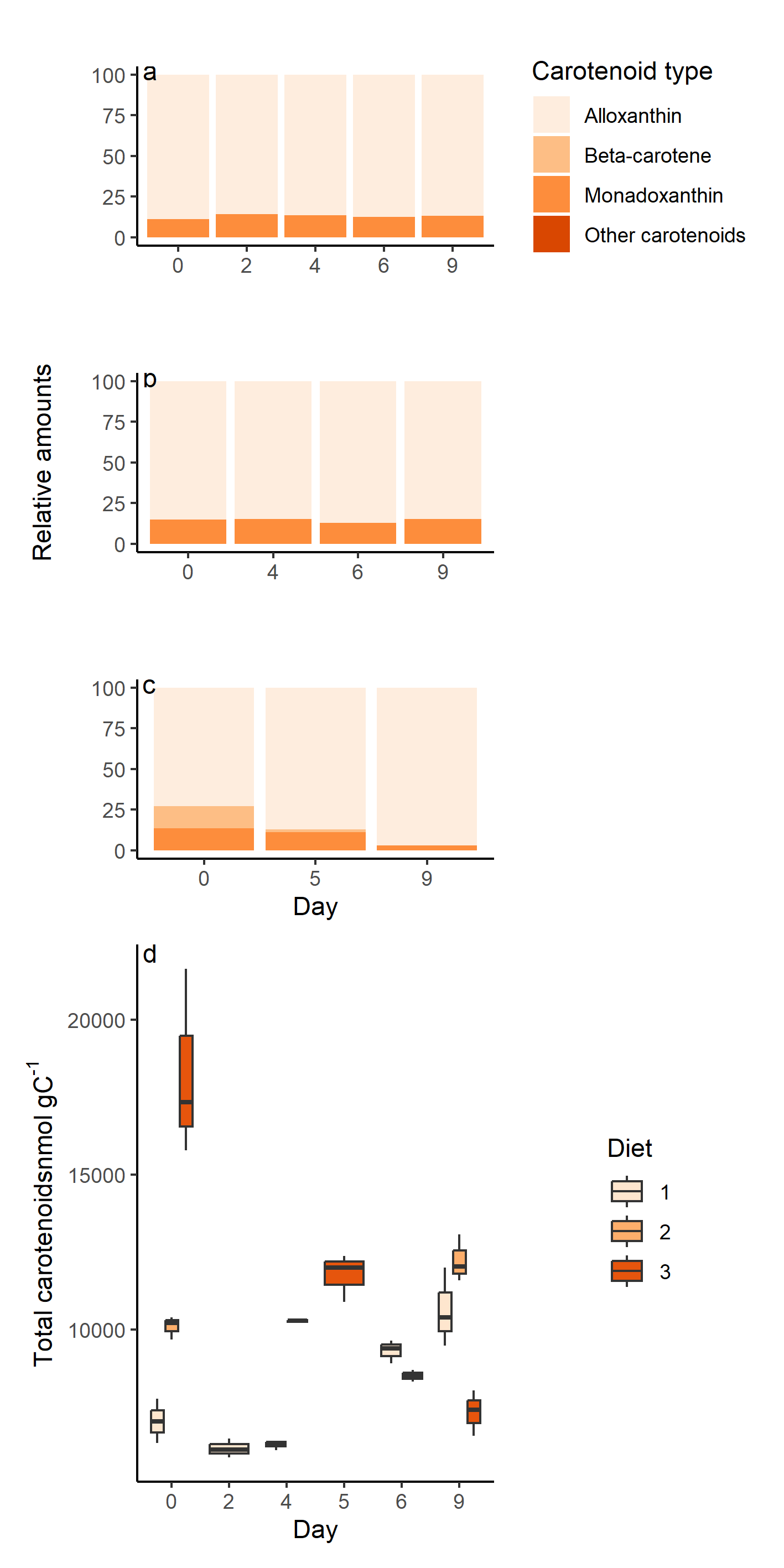


S1 Fig. S1. Relative proportions of carotenoids in Diet 1 (a) Diet 2 (b) and Diet 3 (c), and the total carotenoid concentration (d). Alloxanthin was the most common carotenoid followed by monodoxanthin. β-carotene was only present in Diet 3. Other carotenoids included trace amounts of zeaxanthin, neoxanthin, violaxanthin, and lutein.


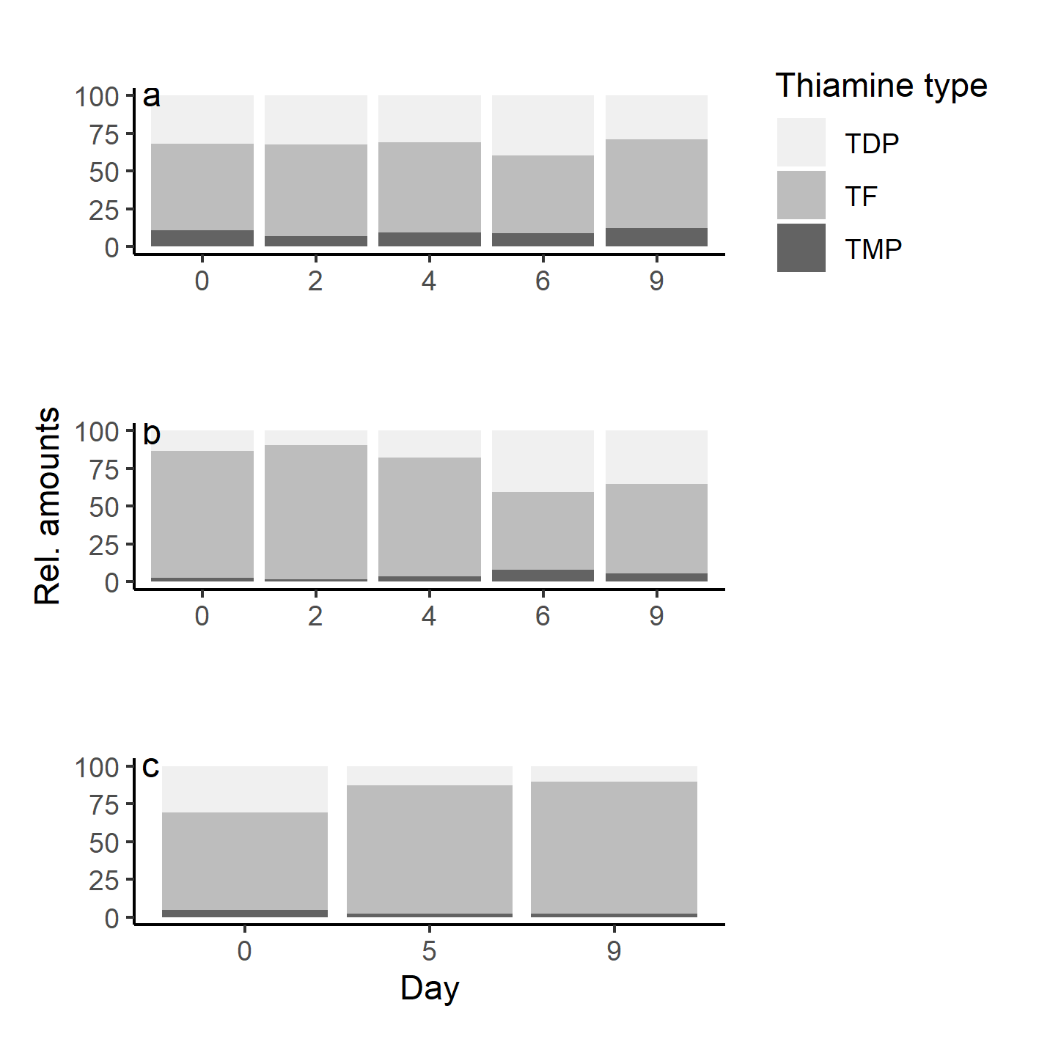


S1 Fig. S2. Relative proportions of thiamine vitamers in Diet 1 (a), Diet 2 (b) and Diet 3 (c). TF was the most abundant vitamer followed by TDP and TMP in the three diets.


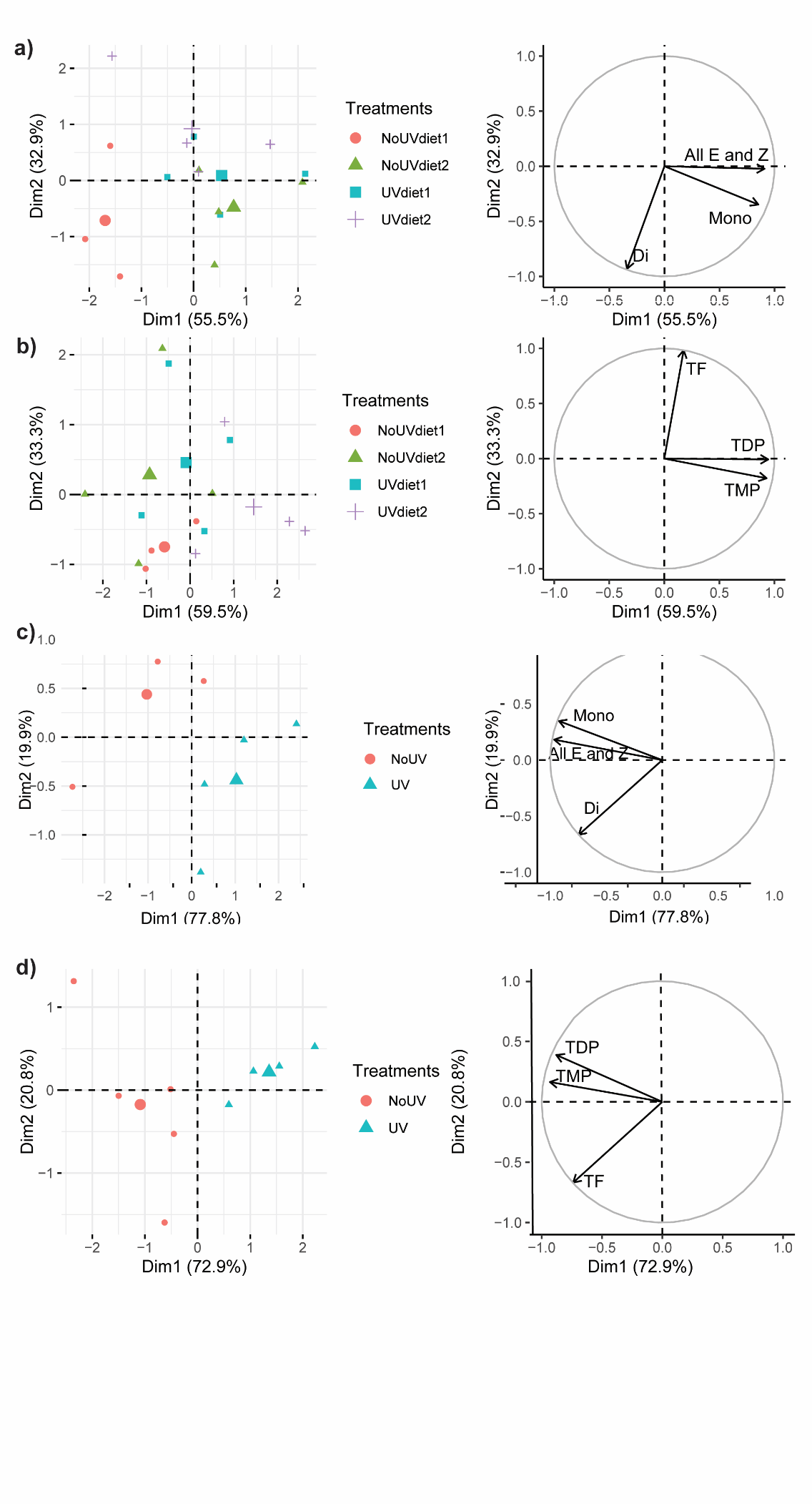


S1 Fig. S3. Illustration of mixtures of astaxanthin in copepods using Diets 1-2 (subpanel a) and Diet 3 (subpanel c). Subpanel b and c illustrate the mixture of thiamine vitamers in copepods using diets 1-2 (b) and diet 3 (d).
